# Hypofractionated Irradiation Suppressed the Off-Target Mouse Hepatocarcinoma Growth by Inhibiting Myeloid-Derived Suppressor Cell-Mediated Immune Suppression

**DOI:** 10.3389/fonc.2020.00004

**Published:** 2020-02-11

**Authors:** Junying Chen, Zeng Wang, Yuxiong Ding, Fei Huang, Weikang Huang, Ruilong Lan, Ruiqing Chen, Bing Wu, Lengxi Fu, Yunhua Yang, Jun Liu, Jinsheng Hong, Weijian Zhang, Lurong Zhang

**Affiliations:** ^1^First Affiliated Hospital of Fujian Medical University, Fuzhou, China; ^2^Fujian Key Laboratory of Cancer Immunotherapy and Key Laboratory of Radiation Biology, Fujian Province Universities, Fuzhou, China; ^3^Department of Otolaryngology, Fujian Provincial Geriatric Hospital, Fuzhou, China; ^4^Fujian Medical University Cancer Hospital, Fujian Cancer Hospital, Fuzhou, China

**Keywords:** *in situ* tumor vaccine, high-dose low-fraction radiation, myeloid-derived suppressor cells, negative immune breaker, hepatocellular carcinoma

## Abstract

**Background:** Stereotactic radiotherapy treats hepatocellular carcinoma (HCC) at different stages effectively and safely. Besides its direct killing of cancer cells, radiotherapy stimulates host immunity against hepatoma. However, the role of myeloid-derived suppressor cells (MDSCs) in on-target and off-target anti-HCC effects induced by hypofractionated irradiation (IR) is unclear.

**Methods and Materials:** Hepa1-6 and H22 allogeneic transplanted tumors on hind limbs of C57BL/6 and Institute of Cancer Research (ICR) mice, respectively, were irradiated with 0, 2.5, 4, 6, or 8 Gy/fraction until the total dose reached 40 Gy. The off-target effect induced by the IR was investigated by subsequently inoculating the same HCC cells subcutaneously on the abdomen. MDSCs in peripheral blood and tumor tissues were measured by flow cytometry or immunofluorescence microscopy analysis. IL-6, regulated on activation normal T cell expressed and secreted (RANTES), and granulocyte colony-stimulating factor (G-CSF) in irradiated mouse plasma and hepatoma cell cultures were measured with ELISA kits. Conditioned media (CM) from irradiated HCC cell cultures on bone marrow cell differentiation and MDSC proliferation were examined by co-culture and flow cytometry.

**Results:** Our study showed that the IR of primarily inoculated HCC on hind limbs created an “*in situ* tumor vaccine” and triggered the antitumor immunity. The immunity was capable of suppressing the growth of the same type of HCC subcutaneously implanted on the abdomen, accompanied with reduced MDSCs in both blood and tumors. The decreased MDSCs were associated with low plasma levels of IL-6, RANTES, and G-CSF. The cytokines IL-6 and RANTES in the CM were lower in the high single IR dose group than in the control groups, but G-CSF was higher. The CM from high single-dose IR-Hepa1-6 cell culture reduced the differentiation of C57BL/6 mouse bone marrow cells into MDSCs, whereas CM from high single-dose IR-H22 cells reduced the proliferation of MDSCs, which might be due to the decreased p-STAT3 in bone marrow cells.

**Conclusions:** The hypofractionated IR on transplanted tumors at the primary location exerted a strong antitumor effect on the same tumor at a different location (off target). This abscopal effect is most likely through the reduction of MDSCs and decrease of IL-6, RANTES, and G-CSF.

## Introduction

Myeloid derived suppressor cells (MDSCs), a group of high-heterogeneity immune-negative regulating cells, have two subgroups: granulocytic MDSC (PMN-MDSC) and monocytic MDSC (M-MDSC) with their own functions (1). In pathological conditions (such as an infection and an autoimmune disease), overproduced inflammation molecules and overstimulated proliferation, and differentiation of immune cells could be restrained by MDSCs to keep the reaction under control and to balance immune response and host's homeostasis (2).

The traditional and new treatments are unsatisfactory for hepatocellular carcinoma (HCC) (3–5). Recently, stereotactic body radiotherapy (SBRT) has emerged as a preferred regimen for HCC owing to its effectiveness and safety (6, 7). Besides its direct killing of tumor cells, the irradiation (IR) also induces immune reactions that kill metastatic hepatoma tumor cells (8). Radiotherapy (RT) enhances the release of tumor-associated antigens (TAAs), creates damage-associated molecular patterns (DAMPs), and stimulates the immunomodulatory cell surface molecules, resulting in a manifestation “*in situ* vaccine” and antitumor immune response (9–11). The IR-targeted tumor could suppress the off-target tumors (tumors at locations away from the irradiated location) (12). This abscopal effect might relate to a fact that the IR turns on the body's antitumor immune response by up-regulating the tumor immunogenicity, which has been well summarized by Demaria and his colleagues (13, 14, 33). However, cellular, molecular, and immunological mechanisms of this off-target effect are not well-studied. Because MDSCs have a significant inhibitory effect on the immunity against malignancies during the development and progression, it is desired to understand the role of MDSCs in IR-induced on-target and off-target antitumor effects.

The alterations of MDSCs could be triggered by different IR regimens (15). IR induces the MDSCs, dendritic cells (DCs), macrophages, and other cells in the lymph nodes surrounding the tumor (16, 17), and affects the recruitment and redistribution of MDSCs in tumor (16, 18, 19). Crittenden et al. found that a total dose of 20 Gy (~6 Gy × 3) given to 4T1- or Panc02 tumor-bearing mice could increase infiltrated MDSCs but decreased blood MDSCs significantly (20). Deng et al. reported that after a single-dose 12-Gy IR, the decreased MDSCs negatively correlated with the increased CD8^+^ cells (21). IR also reduces MDSC levels, which requires high-dose ablative IR rather than multiple lower-dose treatments (22). Thus, MDSCs play an important role in the outcome of tumor RT (23). However, so far, there is no consensus about the best way of IR to fully utilize MDSCs in the RT owing to the lack of systematical comparison study of the IR effects on MDSCs.

We hypothesize that hypofractionated IR of primary tumor generated “*in situ* vaccine,” which could suppress the off-IR-target tumor growth by reducing MDSCs in blood and tumor tissues. To prove this hypothesis, MDSCs in two IR HCC models and the consequent abscopal effects on off-target tumor growths were examined. In addition, MDSCs regulated inflammation molecules [granulocyte colony-stimulating factor (G-CSF), IL-6, and regulated on activation normal T cell expressed and secreted (RANTES)], and their effects on differentiation and proliferation of MDSCs were also explored.

## Materials and Methods

### Cell Culture

Hepa1-6 cells [murine HCC, from American Type Culture Collection (ATCC), Manassas, USA] were cultured in Dulbecco's modified Eagle medium (DMEM); and H22 cells (murine HCC, from Bio-Rad Life Sciences Development Co., Ltd. Beijing, China) were cultured in Roswell Park Memorial Institute (RPMI) 1640 in 37°C in a humidified incubator with 5% CO_2_. The media contained 10% fetal bovine serum (FBS) and 100 U/ml of penicillin and 100 μg/ml of streptomycin. The culture media, FBS, and antibiotics were purchases from Thermo Fisher Scientific (USA).

### Animal Models

**Figure 1:**
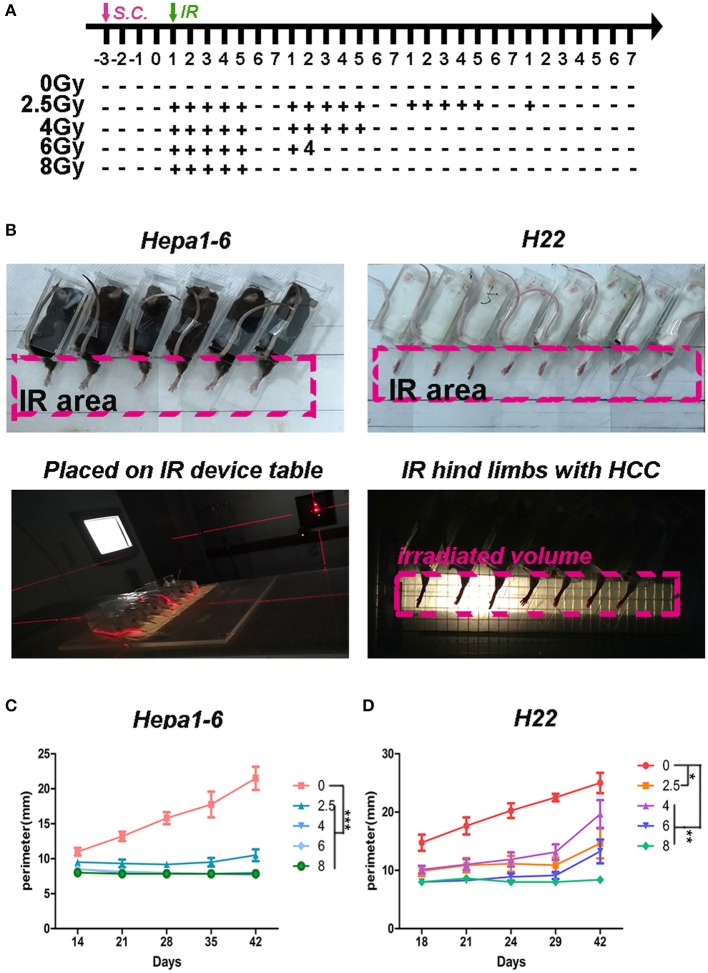
Hypofraction irradiation (IR) significantly improved the local control of hepatocellular carcinoma (HCC) growth in mice. **(A)** Schedule of irradiation: after the Hepa1-6 or H22 allogeneic tumors in C57BL/6 or Institute of Cancer Research (ICR) mice were established, the IR was conducted with different doses (2.5, 4, 6, or 8 Gy) per fraction every day during weekdays until the total dose reached 40 Gy. The images of irradiation procedure of Hepa1-6/C57BL/6 and H22/ICR mouse models are shown **(B)**. The tumors in the hind limbs of mice were fixed in a special device and placed on the 1-cm tissue equivalent compensator and exposed to the IR (voltage, 6 MV; direction, 180°; dose rate, 5 Gy/min; irradiated volume, 36 × 4 cm; distance from source to skin, 100 cm) of linear accelerator (CL/1800, Varian Medical System Inc, USA). The tumor growth in hind limbs was measured by circumference ruler once a week for C57BL/6 mice or twice a week for ICR mice **(C,D)**. The differences of intra-groups were analyzed by one-way ANOVA test followed by Tukey's honestly significant difference (HSD) test in Kruskal–Wallis test. *p < 0.05, **p < 0.01, and ***p < 0.0001. (*n* = 30, 6 mice/group, 5 groups in Hepa1-6/C57BL/6 model; *n* = 40, 8 mice/group, 5 groups in the H22/ICR models).

C57BL/6 and Institute of Cancer Research (ICR) mice (8-week-old pathogen-free female mice) were purchased from Slaccas Experimental Animal LLC (Shanghai, China). Hepa1-6 cells and H22 (1 × 10^6^ cells/in 0.1 ml/site) were subcutaneously injected into hind limbs of C57BL/6 mice and ICR mice, respectively. Three days after the inoculation, the tumors were established as they were touchable (size about 2 mm^3^). Each type of the tumor-bearing mice was randomly divided into five groups (Hepa1-6/C57BL/6, 6 mice/group; and H22/ICR, 8 mice/group) for different IR doses/fractions (0, 2.5, 4, 6, or 8 Gy) with 40-Gy total dose. The IR schedule was as indicated in Figure 1B. Briefly, the mice were immobilized in a special device. The hind limbs bearing the HCC were stretched out, fixed on rear supporter as part of the device, placed on the 1-cm tissue equivalent compensator, and exposed to the IR (voltage, 6 MV; direction, 180°; dose rate, 5 Gy/min; irradiated volume, 36 cm × 4 cm; distance from source to skin, 100 cm) of linear accelerator (CL/1800, Varian Medical System Inc, USA).

The second tumor challenge of Hepa1-6 cells or H22 (1 × 10^6^ cells/0.1 ml/site) was subcutaneously injected on the abdomen 3 days after the entire IR was completed.

The tumor volume of the hind limbs was evaluated by measuring with circumference ruler owing to the unclear tumor boundary. The volume of the second tumor challenge was measured with vernier caliper weekly for Hepa1-6/C57BL/6 models and twice a week for H22/ICR models; and the tumor volume was calculated as length × width^2^/2.

All mice had *ad libitum* access to standard diet and water. The growth of subsequently inoculated tumors and animal well-being were closely monitored. All animal experiments were approved by Fujian Medical University Institutional Animal Ethical Committee (FJMU IACUC 2018-027).

### Flow Cytometric Analysis for Blood Myeloid-Derived Suppressor Cells

Blood sample of mice was collected from the tail vein. After red blood cells were lysed with ACK lysis buffer (Sigma, USA), white blood cells (WBCs) were stained with fluorescein isothiocyanate (FITC)- or allophycocyanin-conjugated anti-mouse CD11b and phycoerythrin (PE)-conjugated anti-mouse Gr1 on ice for 45 min, and then the 7-aminoactinomycin (7AAD) (BioLegend, USA) was added for another 15 min. After being washed three times, the cells were analyzed by Accuri C6 flow cytometer (Becton Dickinson, USA). The negative 7AAD cells were gated for the live cells. Forward vs. side scatter (FSC vs. SSC) was used to gate the subpopulations of monocytes or neutrophils. It has been proved that the morphology of M-MDSCs (CD11b^+^Ly6G^−^Ly6C^+^ or CD11b^+^Ly6G^−^Ly6C^hi^) was mononuclear and that of PMN-MDSCs (CD11b^+^Ly6G^+^Ly6C^−^ or CD11b^+^Ly6G^+^Ly6C^lo^) was multinuclear (24). We used FSC/SSC-gated method to distinguish the mononuclear cells from granulocytes and to determine the MDSCs in the two groups by CD11b and Gr1 antibodies. The two groups of cells were stained with DAPI (BioLegend, USA) after being sorted by FACSAria™ (Becton Dickinson, USA). The nuclear morphology was observed using a fluorescence microscope (Olympus, Japan). In the FSC/SSC-gated mononuclear cells, the CD11b^+^Gr1^+^ cells were recognized as M-MDSCs. In the FSC/SSC-gated granulocytes, the CD11b^+^Gr1^+^ cells were recognized as PMN-MDSCs. The percentage of CD11b^+^Gr-1^+^ MDSCs was calculated in gated monocytes or neutrophils using FlowJo7.6 software.

### Immunofluorescence Analysis for Infiltrated Myeloid-Derived Suppressor Cells in Tumor Tissues

For infiltrated MDSCs in tumor tissues, tumor tissues from the mice were fixed in 10% neutralized formalin overnight and embedded in paraffin blocks [formalin fixed paraffin embedded (FFPE)]. FFPE slides that are 3–4 μm thick were cut from the blocks. The slides were deparaffinized with xylene, rehydrated with gradual alcohols, and incubated in 0.01 M of sodium citrate buffer (pH 6.0) in a 95°C water bath for 15 min for antigen retrieval. After being blocked with 5% FBS in 0.1% PBST (Triton X-100–PBS) for non-specific binding sites, the slides were incubated with FITC-conjugated anti-mouse CD11b and PE-conjugated anti-mouse Gr1 (BioLegend, USA) overnight at 4°C. For MDSC density, the CD11b^+^Gr1^+^ yellow area in random 0.42-mm^2^ field within the tumors was measured and averaged. Images were acquired using a fluorescence microscope (Olympus, Japan). Quantification of fluorescent signals was performed using ImageJ software.

### *In vitro* Radiation Response Assay

Hepa1-6 or H22 cells were seeded in 6-cm plates, each with 4 × 10^5^ cells. After being cultured for 24 h, the cells were placed on the 1-cm tissue equivalent compensator and exposed to the IR (voltage, 6 MV; direction, 180°; dose rate, 5 Gy/min; irradiated volume, 10 cm × 10 cm; distance from source to skin, 100 cm) of linear accelerator (CL/1800, Varian Medical System Inc, USA) at different single doses (0, 2.5, 4, 6, or 8 Gy). At the indicated time points, the conditioned media (CM) were collected, and cell debris in CM was removed by centrifugation. The cells in the plates were washed with PBS, harvested, and frozen at −80°C for subsequent analyses.

### *In vitro* Induction of Myeloid-Derived Suppressor Cells

Protein concentrations in the collected CM from the aforementioned irradiated Hepa1-6 or H22 cultures were determined by bicinchoninic acid (BCA) assay (Beyotime, China) and adjusted to the final concentration of 1 mg/ml. Bone marrow cells isolated from C57BL/6 or ICR mice were adjusted to the final density of 2.5 × 10^6^/ml in culture media in the presence of 10 μg/ml of granulocyte-macrophage colony-stimulating factor (GM-CSF) (BioLegend, USA) or the CM from irradiated Hepa1-6 or H22 cells. Three days after the culture, the percentage of CD11b^+^Gr1^+^ MDSCs was measured with flow cytometry (FCM). The proliferation of MDSC was detected by 5,6-carboxyfluorescein diacetate (CFSE) staining.

### Cytokine Assay

IL-6, G-CSF, and RANTES in the experimental mouse plasma and the CM collected from irradiated H22 or Hepa1-6 cells cultured *in vitro* were quantified using Mouse Cytokine ELISA Kit (MULTI SCIENCES, China) following the manufacturers' instructions.

### Western Blot for p-P65 and p-STAT3

All steps for Western blot were consistent with the published literature (25). Briefly, the irradiated HCC cells were lysed with TNE buffer (10 mM of Tris-HCl, 150 mM of NaCl, 1 mM of EDTA, and 0.5% NP40, pH 7.5). Protein concentrations in the lysates were measured and adjusted to 2 mg/ml. The tumor cell lysates were mixed with 4 × loading buffer [40 mM of Tris-HCl, 200 mM of DTT, 4% sodium dodecyl sulfate (SDS), 40% glycerol, and 0.032% bromophenol blue, pH 8.0]. The mixtures were run on SDS–polyacrylamide gel electrophoresis (SDS-PAGE) gel with 4% stacking gel and 10% separating gel. After being run, separated proteins in the gels were then transferred to nitrocellulose membranes for standard Western blot assay. Target protein bands on the membranes were detected with specific antibodies and developed with Thermo Pierce ECL kit. The results were quantified on FluorChem E exposure device (ProteinSimple, USA).

### Statistical Analysis

Quantitative data are presented as average ± standard deviation (SD) unless otherwise indicated. Statistical significance was determined with the one-way ANOVA test followed by Tukey's honestly significant difference (HSD) test with ranks for a multiple-group comparison. The correlation between *in situ* irradiated tumor sizes and the percentage of MDSCs in peripheral blood mononuclear cell (PBMC) of two HCC tumor-bearing models were determined with Pearson correlation with dose as control variate followed by statistical significance set to *p* < 0.05. A statistical analysis of the differences between groups was performed with GraphPad Prism 5.

## Result

### Hypofractionated Irradiation Improved the Local Control of Hepatocellular Carcinoma in Mice

To test what dose per fraction and how many fractions within the clinically used SBRT produce the best antitumor effect, a 40-Gy total ablative dose with different doses/fractions was tested in the two allogeneic HCC mouse models described in the *Materials and Methods*. The fractional radiation schedule is indicated in Figure 1A. The IR procedure and the quality control of the IR procedure are shown in Figure 1B. The IR suppression of the on-target tumor growth was dose dependent (Figures 1C,D). The higher the dose/low fraction used, the greater the suppression of the tumor growth obtained. In Hepa1-6/C57BL/6 model, higher than 4 Gy/fraction of hypofractionated IR (×10) could completely inhibit the tumor growth and 8 Gy/fraction of the hypofractionated IR (×5) completely suppressed tumor growth in the H22/ICR models for 42 days.

### Effects of Irradiation-Induced *in situ* Vaccine on the Growth of Off-Target Tumors

**Figure 2:**
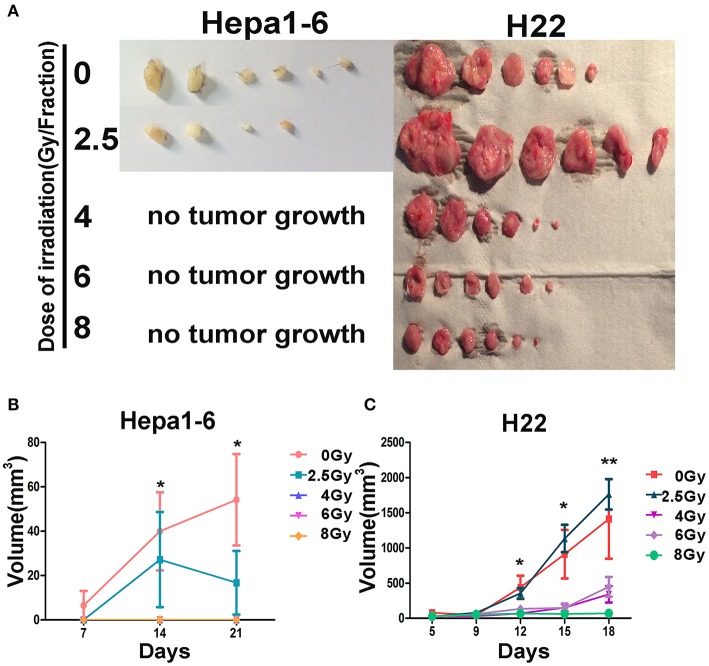
Effectiveness of the on-target vaccine created by hypofraction irradiation (IR) on off-target tumors was dose dependent. The allogeneic tumors on mouse hind limbs underwent different IR doses/fraction to create *in situ* vaccines. The antitumor immunity elicited by *in situ* vaccines was evaluated by observing the growth of the second tumor challenge on abdomen in both Hepa1-6/C57BL/6 and H22/Institute of Cancer Research (ICR) models. The second tumor challenge volume was measured weekly for Hepa1-6/C57BL/6 models and twice a week for H22/ICR models; and the tumor were calculated as length × width^2^/2. Results showed that all irradiated on-target tumors had an antitumor immunity effect on the second inoculated off-target tumors **(A)**, except for H22 tumors irradiated with 2.5 Gy/fraction, in which the off-target tumors grew bigger than controls. The antitumor immunity effect of the *in situ* vaccines on the second inoculated off-target tumors (abscopal effect) was dose per fraction dependent **(B,C)**. The higher the dose per fraction (>4 Gy) used, the stronger the antitumor immunity against the second tumor challenge developed. *P < 0.05, **P < 0.01.

To test whether irradiated tumors formed by Hepa1-6 or H22 cells on one hind limb of each mouse could serve as “IR-induced *in situ* vaccine” and trigger an immune response to exert “abscopal effect,” the same tumor cells were subsequently inoculated under the abdominal skin. Results showed that all irradiated on-target tumors had an antitumor immunity effect on the second inoculated off-target tumors (Figure 2), except for H22 tumors irradiated with 2.5 Gy/fraction, in which the off-target tumors grew bigger than controls. With the same 40-Gy total dose, the high dose and low fraction of 8 Gy × 5 groups had the strongest immune response and the best abscopal effect than had other low dose/fraction groups in both HCC-bearing mouse models.

### Hypofractionated Irradiation Reduced Blood Myeloid-Derived Suppressor Cells, Which Was Positively Correlated With the Growth of the Tumors

**Figure 3:**
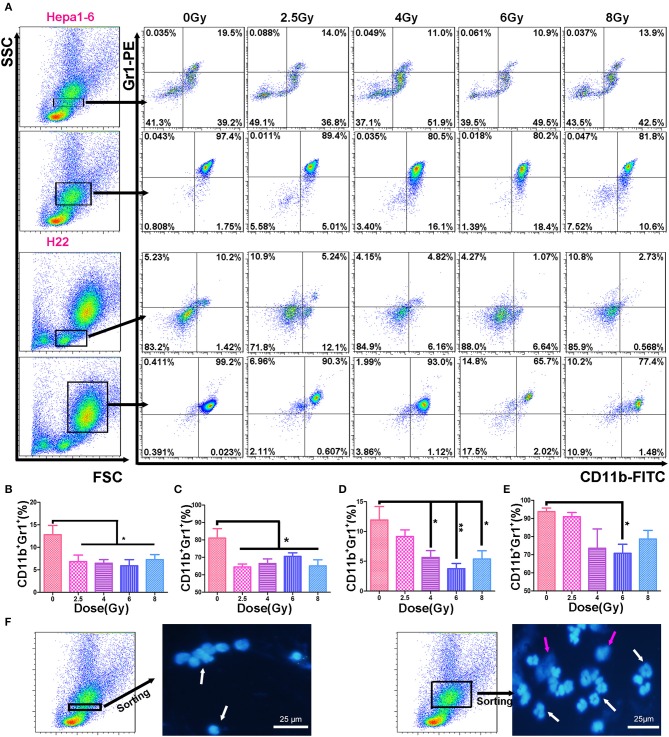
Hypofraction irradiation (IR) reduced the percentage of myeloid-derived suppressor cells (MDSCs) in peripheral blood. On day 3 after a 40-Gy total IR, MDSCs (CD11b^+^Gr1^+^) in mouse peripheral blood were measured with flow cytometry (FCM) in both Hepa1-6/C57BL/6 and H22/Institute of Cancer Research (ICR) mouse models **(A)**. The percentages of MDSCs within monocyte population (the first and third rows of FCM charts, **A**) and granulocyte population (the second and fourth rows) in Hepa1-6/C57BL/6 (the first and second rows) and H22/ICR (the third and fourth rows) mouse models were compared among the groups with different doses of fractional IR. There were significant differences in monocyte-like MDSCs **(B,D)** and granulocyte-like MDSCs **(C,E)** between irradiated groups and unirradiated control group in both animal models [**(B, C)** from Hepa1-6/C57BL/6; **(D, E)** from H22/ICR mouse model] (*p < 0.05 and **p < 0.01). The nuclear morphological differences between the monocyte-gated MDSCs and granulocyte-gated MDSCs were sorted by flow cytometry and observed after DAPI staining. Images were acquired using a fluorescence microscope (Olympus, Japan). Magnification 400× white ↑ for target nuclear and red ↑ for impurities in the background **(F)**.

The immunosuppressive tumor microenvironment has been believed to be not only one of the key factors stimulating tumor progression but also a strong obstacle for efficient tumor therapy (26). MDSCs as heterogeneous immunosuppressive cells develop and expend during the tumor progression (27). To determine the alteration of MDSCs in peripheral blood during the progression of allogeneic HCC tumors in mouse models, the double-stained (CD11b^+^Gr1^+^) MDSCs were measured in two populations: mononucleocytes and granulocytes with FCM (Figure 3A). Results showed that the IR decreased peripheral blood MDSCs in both animal models. There was a significant difference in the percentages of monocyte-like MDSCs (Figures 3B,D) and granulocyte-like MDSCs (Figures 3C,E) between irradiated groups and unirradiated control group. There was no significantly difference in the MDSC percentage among the irradiated groups with different doses/fraction in a C57BL/6 mouse model. There was a tendency that increasing dose/fraction radiation (except for 8 Gy/fraction) could increase its inhibitory effect on MDSCs in peripheral blood. At the high dose/fraction (≥4 Gy/fraction), the magnitude of MDSCs reduction was greater in C57BL/6 mice than in ICR mice.

**Figure 4:**
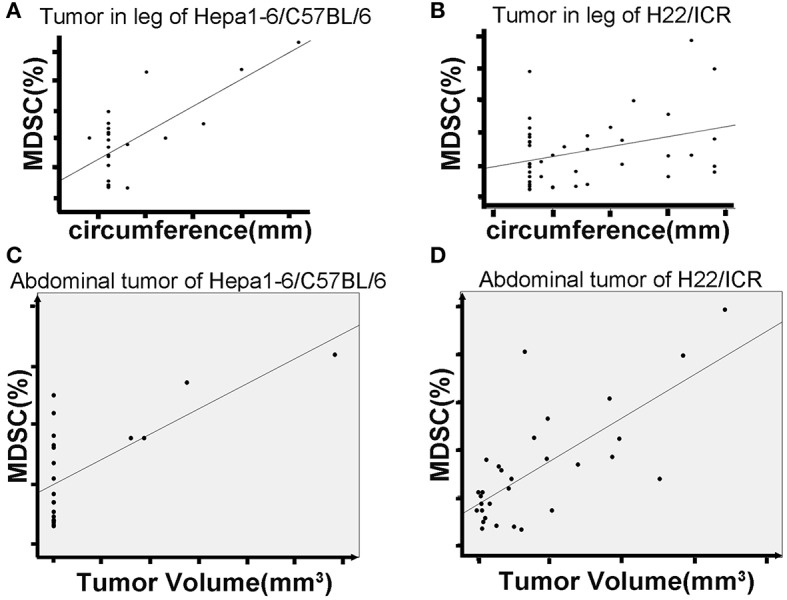
Hypofraction irradiation (IR) reduced blood MDSCs, which was positively correlated with the growth of the tumors. The mice were subcutaneously injected with hepatocellular carcinoma (HCC) cells Hepa1-6 and H22 on one hind limb of each mouse. Three days after the inoculation, the inoculated site was irradiated according to the schedule in Figure 1A. The peripheral blood of the mice was collected from the tail vein. The growth of allogeneic tumors was closely monitored. MDSCs in the blood were measured by flow cytometry. Results showed that the size of on-target tumors is positively correlated with the percentage of MDSCs in total white blood cells (**A** for Hepa1-6 allogeneic tumor and **B** for H22 allogeneic tumor) [*r*^2^ = 0.716, *p* < 0.01 in Hepa1-6/C57BL/6 model and *r*^2^ = 0.332, *p* = 0.032 < 0.05 in H22/Institute of Cancer Research (ICR) models]. There was a positive correlation of blood MDSCs with off-target tumor growth (**C**, **D**, the Pearson *r*^2^ = 0.45, *p* = 0.041 < 0.05 in Hepa1-6/C57BL/6 model; *r*^2^ = 0.529, *p* = 0.003 < 0.01 in H22/ICR models).

Based on the size and nuclear density, the FSC/SSC analysis could further divide CD11b^+^Gr1^+^ MDSCs into monocyte MDSCs (M-MDSCs) *and* granulocyte-like MDSCs (G-MDSCs). Both subpopulations were reduced in mice with IR-treated tumors (Figure 3). The number of MDSCs in peripheral blood was positively correlated with the size of the on-target allogeneic tumors in both Hepa1-6/C57BL/6 (Figure 4A) and H22/ICR (Figure 4B, the Pearson *r*^2^ = 0.716, *p* < 0.01 in Hepa1-6/C57BL/6 model; *r*^2^ = 0.332, *p* = 0.032 < 0.05 in the H22/ICR models).

We also found a positive correlation of blood MDSCs with off-target tumor growth. That is, the higher the number of blood MDSCs, the faster the off-target tumors grew, indicating that MDSCs act as a positive promoter for the tumor growth (Figures 4C,D, the Pearson *r*^2^ = 0.45, *p* = 0.041 < 0.05 in Hepa1-6/C57BL/6 model; *r*^2^ = 0.529, *p* = 0.003 < 0.01 in the H22/ICR models).

### Hypofractionated Irradiation Reduced the Tumor-Infiltrating Myeloid-Derived Suppressor Cells

**Figure 5:**
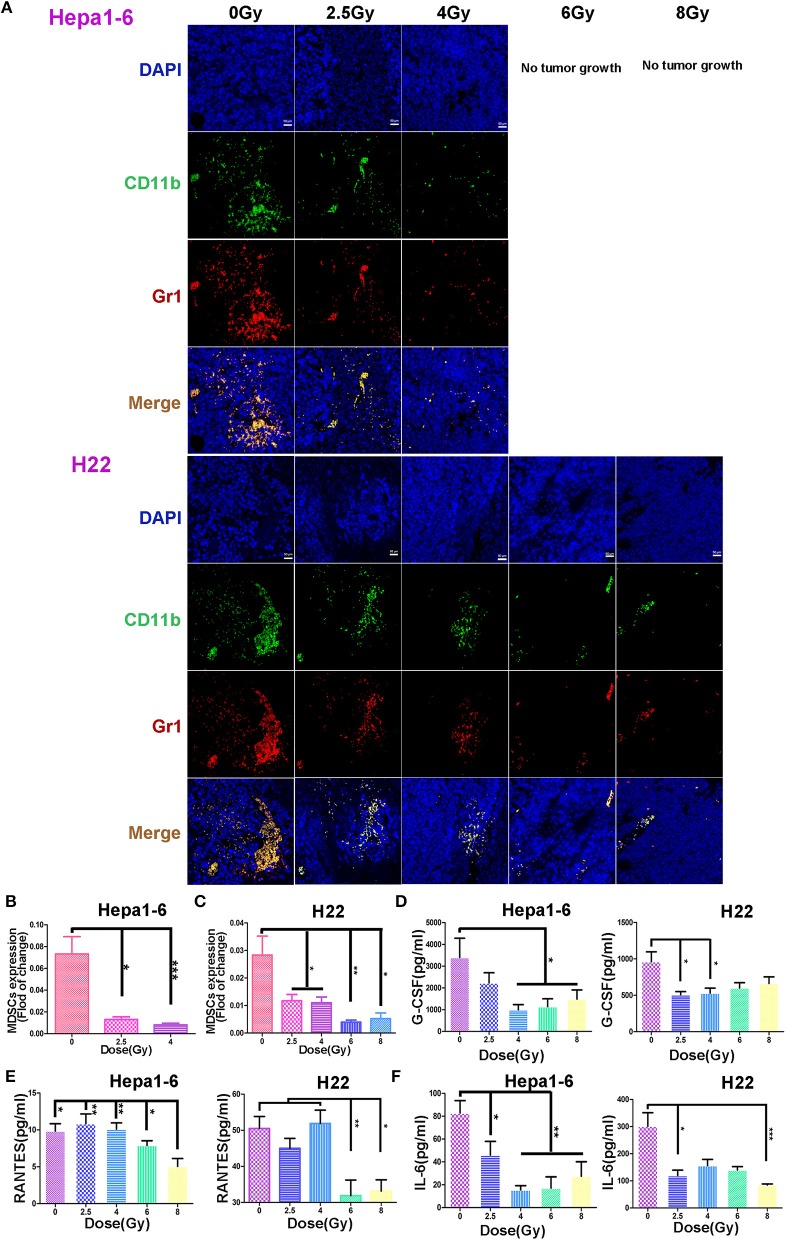
Hypofraction irradiation (IR) reduced the tumor-infiltrating myeloid-derived suppressor cell (MDSC) and plasma granulocyte colony-stimulating factor (G-CSF), IL-6, and regulated on activation normal T cell expressed and secreted (RANTES). The infiltrated MDSCs in allogeneic tumors irradiated with different doses/fraction (2.5, 4, 6, or 8 Gy) and unirradiated tumors were stained with fluorescein isothiocyanate (FITC) anti-mouse CD11b antibody (green), phycoerythrin (PE) anti-mouse Gr1 antibody (red), and DAPI (blue). The infiltrated MDSCs (CD11b^+^Gr1^+^, yellow) decreased with the increase of radiation dose/fraction applied (**A**, magnification, 200×). The MDSCs in Hepa1-6 and H22 tumors irradiated with different IR doses/fraction were compared. Significant differences were found among the groups with different doses/fraction radiation and without radiation (**B** and **C**). Plasma G-CSF, IL-6, and RANTES in peripheral blood of the mice were quantitatively measured with ELISA kits in both Hepa1-6/C57BL/6 and H22/Institute of Cancer Research (ICR) models. Significant differences in these cytokines in the blood were found after IR with different doses/fraction **(D–F)**. *p < 0.05, **p < 0.01, and ***p < 0.0001.

The infiltrated MDSCs in IR-on-target tumor tissues was examined under a fluorescence microscopy after being stained with FITC-labeled anti-mouse CD11b antibody and PE-labeled anti-mouse Gr1 antibody followed by DAPI counterstaining. Figure 5A shows that in non-IR control tumors, there was a large amount of infiltrated MDSCs. The MDSCs were significantly reduced in IR tumors. The higher the IR dose/fraction, the lower the tumor-infiltrating MDSCs, which was more obvious in Hepa1-6 tumors than in H22 tumors after 6 to 8 Gy of IR (Figures 5A–C). The reduction magnitude of infiltrated MDSCs was greater in C57BL/6 mice than in ICR mice. The difference in the infiltrated MDSCs in tumor tissues between unirradiated and irradiated with 2.5–4 Gy/fraction in Hepa1-6 C57BL/6 mice was less significant than in those irradiated with >4 Gy/fraction (Figures 5A,C).

### Hypofractionated Irradiation Reduced the Plasma Cytokines and Chemokines

It is well-known that tumor and host cells in the tumor microenvironment produce the pro-inflammatory mediators that activate MDSCs (28, 29). To test if the alteration of MDSCs is related with IR-induced chemokines and cytokines, the plasma G-CSF, IL-6, and RANTES of each mouse were measured with Mouse Cytokine ELISA kits. The plasma IL-6 was decreased in all tumor IR groups (Figure 5F). The plasma G-CSF was also decreased in irradiated groups, especially in 4–8 Gy C57BL/6 and 2.5–4 Gy ICR groups, with statistical significance (Figure 5D). The plasma RANTES was decreased only in 6- and 8-Gy groups in two mouse models (Figure 5E). These results demonstrate that the IR could decrease the expression of MDSC-related stimulatory cytokines: IL-6, G-CSF, and RANTES.

### Irradiation Caused Hepatocellular Carcinoma Necrosis, Which Was Related to the Activation of NF-κB and to the Alteration of Cytokine Production by Tumor Cells

**Figure 6:**
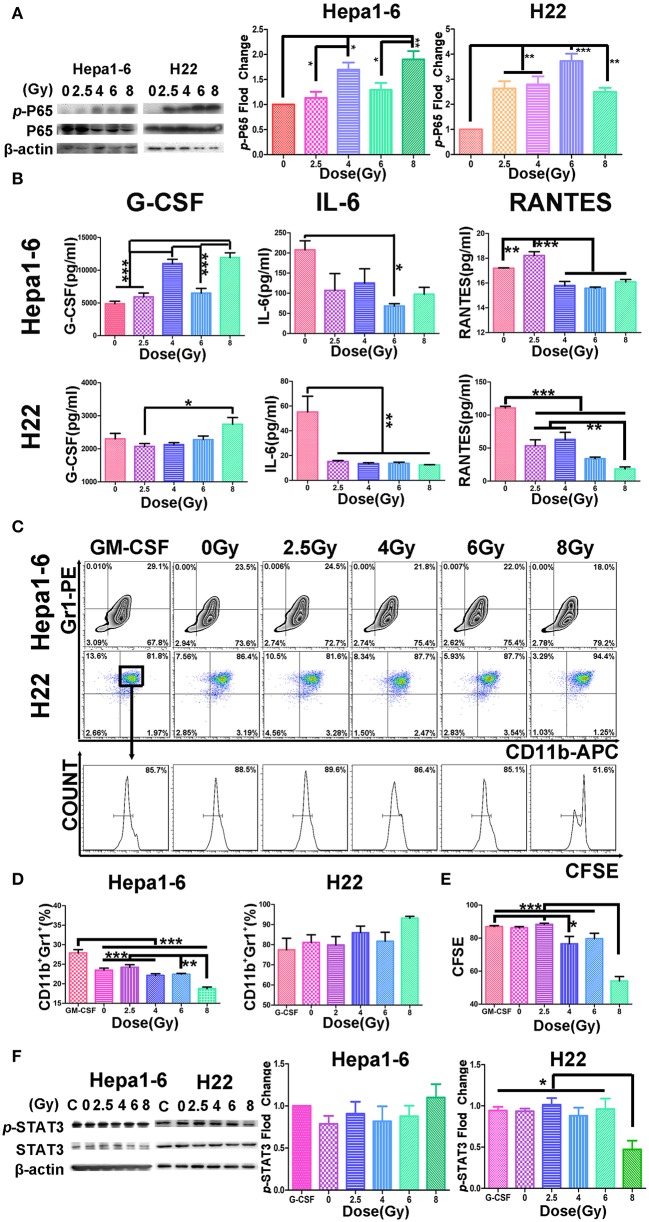
Hypofraction irradiation (IR) increased p-P65, reduced the cytokine secretion from hepatocellular carcinoma (HCC), and inhibited the differentiation or proliferation of MDSCs. **(A)** The phosphorylated P65 increased in Hepa1-6 cells or H22 cells 1 h after IR with different doses of radiation detected by Western blot. **(B)** Two days after Hepa1-6 or H22 cells after IR with different doses (0, 2.5, 4, 6, or 8 Gy), conditioned media (CM) was harvested and the levels of granulocyte colony-stimulating factor (G-CSF), IL-6, and regulated on activation normal T cell expressed and secreted RANTES in CM were measured with ELISA kits. **(C–E)** The bone marrow cells were harvested from C57BL/6 or Institute of Cancer Research (ICR) mice and co-cultured with CM from Hepa1-6 or H22 cells irradiated with different single doses (0, 2.5, 4, 6, or 8 Gy). Three days later, the differentiation of the bone marrow cells into MDSCs or their proliferated was monitored by CD11b/Gr1 staining or 5,6-carboxyfluorescein diacetate (CFSE) assay. By comparing results from these assays, some differences were found among the different groups. *p < 0.05; **p < 0.01 except for the granulocyte-macrophage colony-stimulating factor (GM-CSF) concentration. **(F)** The phosphorylation of STAT3 was also detected by Western blotting; and the phosphorylated and unphosphorylated STAT3 bands on Western blots were scanned and their densities were compared. All cytological experiments were repeated three times. ***P < 0.001.

It is well-known that tumor cell necrosis could be caused by TNF activation of NF-κB accompanied with tissue damage or inflammation (30, 31). To verify the biological response of HCC cells after different doses of IR, we monitored the phosphorylation change of NF-κB and the cytokine secretion from irradiated HCC. Results demonstrated that p-P65 was significantly elevated after IR in both Hepa1-6 and H22 cells (Figure 6A). The elevated p-P65 might relate to the decreased IL-6 and RANTES, whereas the increased G-CSF was significant in the 8-Gy group detected in the CM in both cell lines (Figure 6B).

### Conditioned Media From High Single-Dose Irradiation Altered the Differentiation or Proliferation of Myeloid-Derived Suppressor Cells

To examine whether the cytokines in the IR-induced CM were correlated with the differentiation or proliferation of MDSC, CM of different single-dose IR-HCC were added to the cultures of bone marrow cells from C57BL/6 or ICR mice. Figure 6C shows that CM from high single-dose irradiated Hepa1-6 cells could significantly inhibit the differentiation of bone marrow cells into MDSCs, whereas CM of IR H22 cells did not affect the differentiation of MDSCs but inhibited the proliferation of MDSCs as evidenced by CFSE assay (Figures 6C–E).

We then tested the phosphorylation of STAT3 (p-STAT3) of bone marrow cells transformed into MDSCs *in vitro* triggered by CM from different IR doses of HCC with Western blot (Figure 6F). As expected, the p-STAT3 was significantly reduced in the MDSCs induced with CM from the high-dose irradiated H22 cultures, whereas the p-STAT3 was not changed in the cells treated with IR Hepa1-6 CM.

## Discussion

It has been reported that IR with different dose per fraction schemes could change the tumor immune microenvironment (32, 33) and create “*in situ* vaccine” to induce an effective abscopal effect on remote tumors (off target) (34, 35). This study, using two HCC mouse models, demonstrated that hypofractionated IR was more effective to create the abscopal effect with a high dose per fraction in the same 40-Gy total dose, that is, ≥4 Gy/fraction. The higher the dose/fraction of radiation, the better the inhibition of off-target tumor growth produced.

To reveal the cellular immunological mechanism of the abscopal effect, we focused on the alterations of IR-induced MDSCs (the negative immune breaker), by which hypofractionated IR exerted its off-target effect. It has been well-summarized that RT both promotes and inhibits MDSC function (36). In conventional fractionated IR, there is an increase in MDSCs in both clinical trials and animal models (19, 22). However, high-dose ablative IR reduced the level of MDSCs (22). We found the same results in two HCC tumor-bearing mouse models: (1) the higher the IR dose/fraction, the bigger the off-target effect (Figure 2); (2) the bigger the tumor, the higher the blood MDSCs (both G-MDSCs and M-MDSCs) (Figure 3); (3) the higher IR dose, the less blood MDSCs (Figure 4) and infiltrated tumor MDSCs (Figures 5A–C) found. We also observed that the higher the IR sensitivity, the faster the reduction of tumor size (Figure 1) and MDSCs (Figure 3), and the better the suppression of remote (off-IR-target) tumor (Figure 2). Whether the speed of MDSCs reduction could be utilized as the sensitivity of tumors to IR remains to be further investigated.

Different methods have been used to detect the MDSC subpopulations in blood. Using Ly6G or Ly6C monoclonal antibodies to distinguish the M-MDSCs and PMN-MDSCs was a common method (1). The PMN-MDSCs (CD11b^+^Ly6G^+^Ly6C^−^ or CD11b^+^Ly6G^+^Ly6C^lo^) and M-MDSCs (CD11b^+^Ly6G^−^Ly6C^+^ or CD11b^+^Ly6G^−^Ly6C^hi^) (24) showed that the morphology of M-MDSCs was mononuclear and PMN-MDSCs was multinuclear. In this study, we used the FSC/SSC-gated method to distinguish the mononuclear cells and granulocytes and to determine the MDSCs in the two groups by CD11b and Gr1 antibodies. The two group cells were stained with DAPI after sorting by FACSAria™, and the nuclear morphology was observed using a fluorescence microscope. We proved that M-MDSCs were single nuclear cells and PMN-MDSCs were multinuclear cells (Figure 3F). In the FSC/SSC-gated mononuclear cells, the CD11b^+^Gr1^+^ cells were recognized as M-MDSCs; in the FSC/SSC-gated granulocytes, the CD11b^+^Gr1^+^ cells were recognized as PMN-MDSCs. The different functions of these two MDSC subpopulations in IR patients remain to be for further careful study.

To further reveal the molecular mechanism related to alterations of HCC with hypofractionated IR, we believe that the necrosis signal transduction should be changed. Therefore, the phosphorylation of key factor NF-κB of necrosis signal transduction was monitored. Consistent with others' report (30), we found that p-P65 was significantly elevated after IR in both Hepa1-6 and H22 cells (Figure 6A). The high dose IR of tumor cells also effectively reduced their production of IL-6 and RANTES (Figure 6). The phosphorylation of STAT3 was observed in MDSCs treated with H22 but not Hepa1-6 CM, indicating that the IR released NF-κB in H22 test system to reach the threshold of phosphorylation of STAT3, but not in Hepa1-6 test system. Tumor cell necrosis as triggered by TNF activation of NF-κB was also accompanied with inflammation (31). It might be a reason for G-CSF increase in CM of hypofractionated IR cells. It was reported that cytokines and chemokines, such as IL-6 and RANTES, produced by the tumor cells could promote the generation of MDSCs (37–39), which might explain that CM from high single-dose IR cells lead to less differentiation or proliferation of MDSCs (Figure 6). MDSCs are utilized by tumors to counteract the immune surveillance by suppressing antigen-presenting cells (APCs) such as DCs and macrophages (40), T cells (41), and NK cells (42). When MDSCs as the breaker of immune surveillance are reduced or removed, the APCs, T cells, and NK cells could better exert their effect on the recognition and killing of tumors, which could explain that the MDSCs were positively correlated with tumor size in our two mouse models (Figure 4). The advantage of the hypofractionated IR over surgery is that although surgery simply removes tumors, the “radiation surgery” also creates “*in situ* vaccine” to stimulate the antitumor immunity by removing the MDSC breaker of immune surveillance, a double benefit from IR.

The limitations of this study are as follows: (1) it is a mouse model study, and therefore, the conclusions need to be further confirmed by clinical research; (2) the reduction of MDSCs is one mechanism of the abscopal effect induced by hypofractionated IR, and more underlying mechanisms should be explored.

So far, it is clear that MDSCs, like PD-1, is a negative breaker of immune surveillance. Reduction or removal of MDSCs could be a new strategy for effective treatment of cancers. In fact, several agents have been found to reduce the proliferation of MDSCs or to target MDSCs' trafficking in mouse and human tumors, for example, the inhibitor of CXCR2 (43), the CXCR4 antagonist AMD3100 (44), and the chemotherapeutic drugs doxorubicin (45), sunitinib (tyrosine kinase inhibitor) (46), and Avastin (VEGF-specific monoclonal antibody) (47). Whether the combination of drugs inhibiting MDSC proliferation with hypofractionated IR could enhance the efficacy of antitumor and antimetastasis drugs needs to be studied.

## Conclusion

The hypofractionated (4–8 Gy/fraction) IR exerts strong on-target and off-target antitumor effects *via* the reduction of MDSCs and its related IL-6, RANTES, and G-CSF. The alteration of MDSCs could be a potential target for effective RT.

## Data Availability Statement

All datasets generated for this study are included in the article/Supplementary Material.

## Ethics Statement

This study was carried out in accordance with the recommendations of international guidelines and ethical standards. All animal experiments were approved by Fujian Medical University Institutional Animal Ethical Committee (FJMU IACUC 2018-027).

## Author Contributions

JC, LZ, and ZW conceived and designed the experiments. JC, ZW, YD, FH, WH, JL, BW, RL, RC, and LF performed the experiments. JC, JH, and YY analyzed the data. JC wrote the paper. JC, JH, WZ, and LZ revised the paper.

### Conflict of Interest

The authors declare that the research was conducted in the absence of any commercial or financial relationships that could be construed as a potential conflict of interest.

## References

[1] Bronte V Brandau S Chen SH Colombo MP Frey AB Greten TF. Recommendations for myeloid-derived suppressor cell nomenclature and characterization standards. Nat Commun. (2016) 7:12150. 10.1038/ncomms12150 27381735 PMC4935811

[2] Budhwar S Verma P Verma R Rai S Singh K. The yin and yang of myeloid derived suppressor cells. Front Immunol. (2018) 9:2776. 10.3389/fimmu.2018.02776 30555467 PMC6280921

[3] Han K Kim JH. Transarterial chemoembolization in hepatocellular carcinoma treatment: barcelona clinic liver cancer staging system. World J Gastroenterl. (2015) 21: 10327–35. 10.3748/wjg.v21.i36.10327 26420959 PMC4579879

[4] Sakisaka M Haruta M Komohara Y Umemoto S Matsumura K Ikeda T. Therapy of primary and metastatic liver cancer by human iPS cell-derived myeloid cells producing interferon-β. J Hepatobiliary Pancreat Sci. (2017) 24:109–19. 10.1002/jhbp.422 28008721

[5] Omata M Cheng AL Kokudo N Kudo M Lee JM Jia J Asia-Pacific clinical practice guidelines on the management of hepatocellular carcinoma: a 2017update. Hepatol Int. (2017) 11:317–70. 10.1007/s12072-017-9799-9 28620797 PMC5491694

[6] Lin TA Lin JS Wagner T Pham N. Stereotactic body radiation therapy in primary hepatocellular carcinoma: current status and future directions. J Gastrointest Oncol. (2018) 9:858–70. 10.21037/jgo.2018.06.01 30505586 PMC6219983

[7] Rim CH Kim HJ Seong J. Clinical feasibility and efficacy of stereotactic body radiotherapy for hepatocellular carcinoma: a systematic review and meta-analysis of observational studies. Radiother Oncol. (2019) 131:135–44. 10.1016/j.radonc.2018.12.005 30773180

[8] Jeong KY Lee EJ Kim SJ Yang SH Sung YC Seong J. Irradiation-induced localization of IL-12-expressing mesenchymal stem cells to enhance the curative effect in murine metastatic hepatoma. Int J Cancer. (2015) 137:721–30. 10.1002/ijc.29428 25639194

[9] Yasmin-Karim S Bruck PT Moreau M Kunjachan S Chen GZ Kumar R. Radiation and local anti-CD40 generate an effective *in situ* vaccine in preclinical models of pancreatic cancer. Front Immunol. (2018) 9:2030. 10.3389/fimmu.2018.02030 30245691 PMC6137176

[10] Kaur P Asea A. Radiation-induced effects and the immune system in cancer. Front Oncol. (2012) 2:191. 10.3389/fonc.2012.00191 23251903 PMC3523399

[11] Krysko DV Garg AD Kaczmarek A Krysko O Agostinis P Vandenabeele P. Immunogenic cell death and DAMPs in cancer therapy. Nat Rev Cancer. (2012) 12:860–75. 10.1038/nrc3380 23151605

[12] Romano G Marino IR. Abscopal effects observed in cancer radiation therapy and oncolytic virotherapy: an overview. Drugs Today. (2019) 55:117–30. 10.1358/dot.2019.55.2.2903217 30816886

[13] Muraro E Furlan C Avanzo M Martorelli D Comaro E Rizzo A. Local high-dose radiotherapy induces systemic immunomodulating effects of potential therapeutic relevance in oligometastatic breast cancer. Front Immunol. (2017) 8:1476. 10.3389/fimmu.2017.01476 29163540 PMC5681493

[14] Rodríguez-Ruiz ME Vanpouille-Box C Melero I Silvia chiara formenti and sandra demaria. Immunological mechanisms responsible for radiation-induced abscopal effect. Trends Immunol. (2018) 39:644–55. 10.1016/j.it.2018.06.001 30001871 PMC6326574

[15] Solinas C Porcu M Hlavata Z De Silva P Puzzoni M Willard-Gallo K. Critical features and challenges associated with imaging in patients undergoing cancer immunotherapy. Crit Rev Oncol Hematol. (2017) 120:13–21. 10.1016/j.critrevonc.2017.09.017 29198327

[16] Lugade AA Moran JP Gerber SA Rose RC Frelinger JG Lord EM. Local radiation therapy of B16 melanoma tumors increases the generation of tumor antigen-specific effector cells that traffic to the tumor. J Immunol. (2005) 174:7516–23. 10.4049/jimmunol.174.12.7516 15944250

[17] Lee Y Auh SL Wang Y Burnette B Wang Y Meng Y. Therapeutic effects of ablative radiation on local tumor require CD8^+^ T cells: changing strategies for cancer treatment. Blood. (2009) 114:589–95. 10.1182/blood-2009-02-206870 19349616 PMC2713472

[18] Vatner RE Formenti SC. Myeloid-derived cells in tumors: effects of radiation. Semin Radiat Oncol. (2015) 25:18–27. 10.1016/j.semradonc.2014.07.008 25481262

[19] Xu J Escamilla J Mok S David J Priceman S West B. CSF1R signaling blockade stanches tumor-infiltrating myeloid cells and improves the efficacy of radiotherapy in prostate cancer. Cancer Res. (2013) 73:2782–94. 10.1158/0008-5472.CAN-12-3981 23418320 PMC4097014

[20] Crittenden MR Cottam B Savage T Nguyen C Newell P Gough MJ. Expression of NF-κB p50 in tumor stroma limits the control of tumors by radiation therapy. PLoS ONE. (2012) 7:e39295. 10.1371/journal.pone.0039295 22761754 PMC3386283

[21] Deng L Liang H Burnette B Beckett M Darga T Weichselbaum RR. Irradiation and anti-PD-L1 treatment synergistically promote antitumor immunity in mice. J Clin Invest. (2014) 124:687–95. 10.1172/JCI67313 24382348 PMC3904601

[22] Lan J Li R Yin LM Deng L Gui J Chen BQ. Targeting myeloid-derived suppressor cells and programmed death ligand 1 confers therapeutic advantage of ablative hypofractionated radiation therapy compared with conventional fractionated radiation therapy. Int J Radiat Oncol Biol Phys. (2018) 101:74–87. 10.1016/j.ijrobp.2018.01.071 29619980

[23] Wang D An G Xie S Yao Y Feng G. The clinical and prognostic significance of CD14^(+)^HLA-DR^(−/low)^ myeloid-derived suppressor cells in hepatocellular carcinoma patients receiving radiotherapy. Tumour Biol. (2016) 37:10427–33. 10.1007/s13277-016-4916-2 26846107

[24] Bei Jia Chenchen Zhao Guoli Li Yaxian Kong Yaluan Ma Qiuping Wang A Novel CD48-based analysis of sepsis-induced mouse myeloid-derived suppressor cell compartments. Mediators Inflamm. (2017) 2017:7521701 10.1155/2017/7521701 28337051 PMC5346402

[25] Huang F Chen J Lan R Wang Z Chen R Lin J. δ-Catenin peptide vaccines repress hepatocellular carcinoma growth via CD8^+^ T cell activation. Oncoimmunology. (2018) 7:e1450713. 10.1080/2162402X.2018.1450713 30221043 PMC6136873

[26] Lazăr DC Avram MF Romosan I Cornianu M Tăban S Goldi? A. Prognostic significance of tumor immune microenvironment and immunotherapy: novel insights and future perspectives in gastric cancer. World J Gastroenterol. (2018) 24:3583–616. 10.3748/wjg.v24.i32.3583 30166856 PMC6113718

[27] Kumar V Patel S Tcyganov E Gabrilovich DI. The nature of myeloid-derived suppressor cells in the tumor microenvironment. Trends Immunol. (2016) 37:208–20. 10.1016/j.it.2016.01.004 26858199 PMC4775398

[28] Parker KH Beury DW Ostrand-Rosenberg S. Myeloid-derived suppressor cells: critical cells driving immune suppression in the tumor microenvironment. Adv Cancer Res. (2015) 128:95–139. 10.1016/bs.acr.2015.04.002 26216631 PMC4662416

[29] Clappaert EJ Murgaski A Van Damme H Kiss M Laoui D. Diamonds in the rough: harnessing tumor-associated myeloid cells for cancer therapy. Front Immunol. (2018) 9:2250. 10.3389/fimmu.2018.02250 30349530 PMC6186813

[30] Wertz IE O'Rourke KM Zhou H Eby M Aravind L Seshagiri S. De-ubiquitination and ubiquitin ligase domains of A20 downregulate NF-kappaB signalling. Nature. (2004) 430:694–9. 10.1038/nature02794 15258597

[31] Vanden Berghe T Linkermann A Jouan-Lanhouet S Walczak H Vandenabeele P. Regulated necrosis: the expanding network of non-apoptotic cell death pathways. Nat Rev Mol Cell Biol. (2014) 15:135–47. 10.1038/nrm3737 24452471

[32] Mathieu G Richard C Limagne E Boidot R Morgand V Bertaut A Optimized fractionated radiotherapy with anti-PD-L1 and anti-TIGIT: a promising new combination. J ImmunoTher Cancer. (2019) 7:160 10.1186/s40425-019-0634-9 31238970 PMC6593525

[33] Formenti SC Demaria S. Systemic effects of local radiotherapy. Lancet Oncol. (2009) 10:718–26. 10.1016/S1470-2045(09)70082-8 19573801 PMC2782943

[34] Marconi R Strolin S Bossi G Strigari L. A meta-analysis of the abscopal effect in preclinical models: Is the biologically effective dose a relevant physical trigger? PLoS ONE. (2017) 12:e0171559. 10.1371/journal.pone.0171559 28222111 PMC5319701

[35] McKelvey KJ Hudson AL Back M Eade T Diakos CI. Radiation, inflammation and the immune response in cancer. Mamm Genome. (2018) 29:843–65. 10.1007/s00335-018-9777-0 30178305 PMC6267675

[36] Ostrand-Rosenberg S Horn LA Ciavattone NG. Radiotherapy both promotes and inhibits myeloid-derived suppressor cell function: novel strategies for preventing the tumor-protective effects of radiotherapy. Front. Oncol. (2019) 9:215 10.3389/fonc.2019.00215 31001479 PMC6454107

[37] Ngwa W Irabor OC Schoenfeld JD Hesser J Demaria S Formenti SC. Using immunotherapy to boost the abscopal effect. Nat Rev Cancer. (2018) 18:313–22. 10.1038/nrc.2018.6 29449659 PMC5912991

[38] Zhang Y Lv D Kim HJ Kurt RA Bu W Li Y Ma X. A novel role of hematopoietic CCL5 in promoting triple-negative mammary tumor progression by regulating generation of myeloid-derived suppressor cells. Cell Res. (2013) 23:394–408. 10.1038/cr.2012.178 23266888 PMC3587709

[39] Chen HM Ma G Gildener-Leapman N Eisenstein S Coakley BA Ozao J. Myeloid-derived suppressor cells as an immune parameter in patients with concurrent sunitinib and stereotactic body radiotherapy. Clin Cancer Res. (2015) 21:4073–85. 10.1158/1078-0432.CCR-14-2742 25922428 PMC4720266

[40] Gabrilovich DI Ostrand-Rosenberg S Bronte V. Coordinated regulation of myeloid cells by tumours. Nat Rev Immunol. (2012) 12:253–68. 10.1038/nri3175 22437938 PMC3587148

[41] Mazzoni A Bronte V Visintin A Spitzer JH Apolloni E Serafini P. Myeloid suppressor lines inhibit T cell responses by an NO-dependent mechanism. J Immunol. (2002) 168:689–95. 10.4049/jimmunol.168.2.689 11777962

[42] Li H Han Y Guo Q Zhang M Cao X. Cancer-expanded myeloid-derived suppressor cells induce anergy of NK cells through membrane-bound TGF-beta 1. J Immunol. (2009) 182:240–9. 10.4049/jimmunol.182.1.240 19109155

[43] Di Mitri D Toso A Chen JJ Sarti M Pinton S Jost TR. Tumour-infiltrating Gr-1^+^ myeloid cells antagonize senescence in cancer. Nature. (2014) 515:134–7. 10.1038/nature13638 25156255

[44] Benedicto A Romayor I Arteta B. CXCR4 receptor blockage reduces the contribution of tumor and stromal cells to the metastatic growth in the liver. Oncol Rep. (2018) 39:2022–30. 10.3892/or.2018.6254 29436696

[45] Alizadeh D Trad M Hanke NT Larmonier CB Janikashvili N Bonnotte B. Doxorubicin eliminates myeloid-derived suppressor cells and enhances the efficacy of adoptive T-cell transfer in breast cancer. Cancer Res. (2014) 74:104–18. 10.1158/0008-5472.CAN-13-1545 24197130 PMC3896092

[46] van Hooren L Georganaki M Huang H Mangsbo SM Dimberg A. Sunitinib enhances the antitumor responses of agonistic CD40-antibody by reducing MDSCs and synergistically improving endothelial activation and T-cell recruitment. Oncotarget. (2016) 7:50277–89. 10.18632/oncotarget.10364 27385210 PMC5226582

[47] Feng PH Chen KY Huang YC Luo CS Wu SM Chen TT. Bevacizumab reduces S100A9-positive mdscs linked to intracranial control in patients with EGFR-mutant lung adenocarcinoma. J Thorac Oncol. (2018) 13:958–67. 10.1016/j.jtho.2018.03.032 29684573

